# Comparative Hepatotoxicity Assessment of PFOS and Its Alternative 6:2 FTSA in Adult Female Zebrafish

**DOI:** 10.3390/ani16091368

**Published:** 2026-04-29

**Authors:** Wenying Zhang, Yunru Dong, Yanmin Jian, Yazhe Jia, Keyi Yang, Yang Chen, Yuan Cao, Lulu Guo, Shujing Zhang, Dongwu Liu, Qiuxiang Pang, Shuang Jiao

**Affiliations:** School of Life Sciences and Medicine, Shandong University of Technology, Zibo 255000, China

**Keywords:** 6:2 FTSA, PFOS, hepatotoxicity, transcriptomics, adult female zebrafish

## Abstract

As an alternative to perfluorooctane sulfonate (PFOS), 6:2 fluorotelomer sulfonic acid (6:2 FTSA) has been increasingly produced and detected in aquatic environments, yet its toxicological effects in fish remain incompletely characterized. In this study, adult female zebrafish were exposed for 30 days to solvent control (CK), 50 μg/L PFOS (P50), 50 μg/L 6:2 FTSA (F50), and 500 μg/L 6:2 FTSA (F500), respectively. Histopathological analysis revealed that both compounds induced hepatic injury, with the most severe damage observed in the F500 group. Hepatic transcriptomic analysis identified 645, 191, and 85 differentially expressed genes (DEGs) in the P50, F50, and F500 groups versus CK, respectively. Functional enrichment analysis further demonstrated distinct toxic profiles: PFOS at 50 μg/L primarily disrupted pathways related to the cell cycle, DNA replication, and reproduction. In contrast, 50 μg/L 6:2 FTSA predominantly activated PPAR-mediated lipid metabolism pathways, consistent with a “metabolic toxicity” phenotype. Notably, at 500 μg/L, 6:2 FTSA induced the most severe injury accompanied by a distinct transcriptomic signature—characterized by fewer DEGs but a pronounced enrichment of endoplasmic reticulum stress pathways—suggestive of a shift from metabolic perturbation to overwhelming cellular stress. Biochemical analysis confirmed a significant increase in malondialdehyde (MDA) only in the F50 group, supporting oxidative stress-mediated metabolic toxicity. Collectively, these findings demonstrate that 6:2 FTSA is not a safe alternative to PFOS but exhibits a dose-dependent and multifaceted toxicological profile, with high-dose effects indicative of acute cellular stress. This study underscores the need for case-specific, dose-range inclusive risk assessment of emerging PFAS alternatives.

## Simple Summary

Chemicals known as PFAS are widespread environmental pollutants. While some, like PFOS, are being phased out due to their toxicity, their replacements, such as 6:2 FTSA, are less studied. There is concern that these alternatives might also be harmful. We evaluated whether 6:2 FTSA is a safe substitute for PFOS by exposing adult zebrafish for 30 days. Both chemicals caused liver damage, but through distinct mechanisms. PFOS primarily disrupted cell growth and reproduction processes. In contrast, a lower dose of 6:2 FTSA mainly interfered with fat and energy metabolism. At a higher dose, it induced severe liver cell damage and stress. Our results demonstrate that 6:2 FTSA is not a safe alternative to PFOS but presents a different, dose-dependent hazard. This study underscores the need to thoroughly test replacement chemicals to fully understand their environmental risks.

## 1. Introduction

Per- and polyfluoroalkyl substances (PFASs) constitute a broad category of anthropogenic chemicals defined by an alkyl chain in which hydrogen atoms are substantially or entirely replaced by fluorine [1]. This unique structure imparts exceptional properties, including water and stain repellency and high dielectric strength, granting them significant economic value and wide application across industries such as textiles, papermaking, electroplating, and chemicals [2]. Among them, perfluorooctane sulfonate (PFOS) is a prominent representative. PFOS has been detected widely in diverse environmental matrices, including water, air, soil, and biological samples [3,4,5]. Due to its environmental persistence, evidenced by an estimated half-life of ranging from 3.4 to 5.7 years in humans [6], combined with documented toxicity [7] and bioaccumulation potential [7], PFOS was listed in Annex B of the Stockholm Convention on persistent organic pollutants (POPs) in 2009 [8], prompting worldwide restrictions on its production and use.

**Figure 1:**
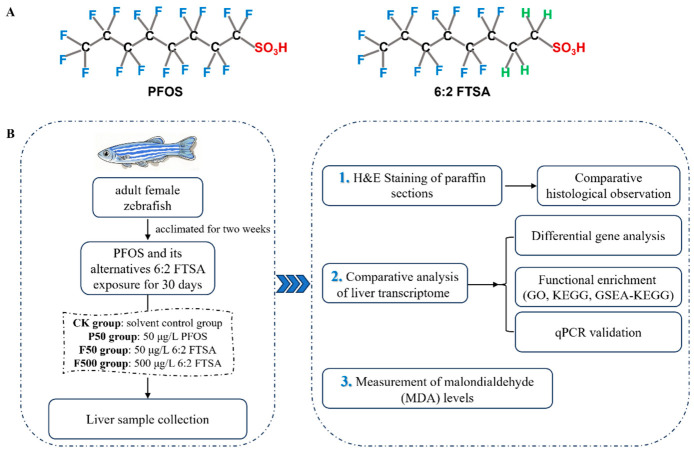
Overview of chemical structures and experimental design. (**A**) Structures of PFOS and 6:2 FTSA. (**B**) Following acclimation, adult female zebrafish were randomly allocated into four groups: control (CK), 50 μg/L PFOS (P50), 50 μg/L 6:2 FTSA (F50), and 500 μg/L 6:2 FTSA (F500). After 30-day exposure, liver tissues were collected for subsequent analyses as outlined in the scheme.

With the progressive phase-out of PFOS, alternative compounds such as 6:2 fluorotelomer sulfonic acid (6:2 FTSA) have been developed [9]. As shown in Figure 1A, unlike the fully fluorinated eight-carbon backbone of PFOS, 6:2 FTSA contains hydrogen substitutions at the first and second carbon positions. This structural modification aims to enhance aqueous solubility, theoretically reducing environmental persistence and toxicity, while preserving key functional properties such as hydrophobicity and oleophobicity [10]. It is primarily used in industrial applications such as aqueous film-forming foams (AFFF) for firefighting [11]. The primary sources of 6:2 FTSA release into the environment likely include its direct use in AFFF at firefighting facilities and the environmental transformation of certain PFAS precursors [9]. Environmental monitoring has revealed the widespread occurrence of 6:2 FTSA in various environmental matrices. In China, for example, 6:2 FTSA has been frequently detected in major rivers and lakes [12], marginal seas [13,14], and semi-enclosed bays such as Laizhou Bay [15]. Notably, its environmental concentrations in some cases have surpassed those of legacy PFOS, raising concern about its safety [14,16]. A striking example occurred in Australia, where a chemical storage facility fire led to acute local contamination, with 6:2 FTSA levels reaching 3000 ng/L in adjacent freshwater [17]. Given its widespread environmental presence, toxicological studies on aquatic organisms such as zebrafish (*Danio rerio*) are essential. Previous toxicological studies on zebrafish have consistently linked 6:2 FTSA exposure to a range of adverse outcomes. During embryonic development, observed effects included immunotoxicity [18] and behavioral alterations [19], although swim bladder inflation remained unaffected [20]. In adult zebrafish (studies not sex-specific), exposure has been associated with abnormal behaviors and manifestations of neurotoxicity [21]. These findings necessitate a careful reevaluation of 6:2 FTSA’s suitability as a safe alternative to PFOS.

Consistent with concerns for liver toxicity, the liver is a recognized target organ for PFOS exposure, with documented hepatotoxic effects in both mammals [22,23] and fish [24,25]. Given the structural similarity between 6:2 FTSA and PFOS, there is reasonable concern that 6:2 FTSA might elicit comparable liver damage. Indeed, studies in mice have shown that 6:2 FTSA can increase liver weight and induce histopathological injury [26]. However, data regarding its hepatic effects in fish remain scarce, creating a critical gap in the ecological risk assessment of this emerging contaminant.

The zebrafish serves as an established model organism for aquatic toxicology [25]. This study focused exclusively on adult female zebrafish to control for the sex as a confounding variable and to address the current lack of hepatotoxicity data for 6:2 FTSA in females. It was designed with two primary objectives: to directly compare the hepatotoxicity of PFOS and its alternative 6:2 FTSA at a comparable concentration, and to delineate the dose–response relationship of the less-characterized 6:2 FTSA. Accordingly, adult female zebrafish were exposed for 30 days to solvent control, 50 μg/L PFOS, 50 μg/L 6:2 FTSA, or 500 μg/L 6:2 FTSA. Histopathological alterations in the liver were analyzed and compared. To further elucidate the underlying mechanisms, comparative transcriptomic profiling of liver tissue was performed, and hepatic malondialdehyde (MDA) levels were quantified.

## 2. Materials and Methods

### 2.1. Chemicals and Reagents

PFOS (CAS No. 2795-39-3) and 6:2 FTSA (CAS No. 27619-97-2), both with a purity of 98%, were purchased from J & K Scientific Ltd. (Beijing, China) and Shanghai Macklin Biochemical Co., Ltd. (Shanghai, China), respectively. Dimethyl sulfoxide (DMSO, CAS No. 67-68-5, purity > 99%) was purchased from Beijing Labgic Technology Co., Ltd. (Beijing, China). Stock solutions (2 g/L) were prepared by dissolving PFOS in DMSO and 6:2 FTSA directly in fish water (pH 7.5 ± 0.5, dissolved oxygen 7.0 ± 0.5 mg/L, conductivity 500 ± 50 μS/cm). All working solutions were then prepared from these stocks. To standardize the vehicle across all exposure groups, DMSO was added to the 6:2 FTSA solutions, ensuring a consistent final concentration of 0.0025% (*v*/*v*) DMSO in the control, PFOS, and 6:2 FTSA groups. All other chemicals used were analytical grade or HPLC grade. The MDA detection kit was procured from Nanjing Jiancheng Bioengineering Institute (Nanjing, China).

### 2.2. Fish Exposure and Sampling

Adult zebrafish (wild-type, AB stain; 4–5 months old) were obtained from Shanghai FishBio Co., Ltd. (Shanghai, China) and acclimatized under for two weeks prior to experimentation. Healthy adult female zebrafish (body weight = 0.156 ± 0.005 g) were randomly assigned to four experimental groups: a solvent control group (0.0025% DMSO; CK group), 50 µg/L PFOS exposure (P50 group), 50 µg/L 6:2 FTSA exposure (F50 group), and 500 µg/L 6:2 FTSA exposure (F500 group). The system was operated semi-statically with three replicates per group *(n* = 20 fish/tank; 8 L solution per tank). The rationale for concentration selection differed between compounds: the PFOS dose was referenced from previous chronic toxicity studies [27,28]. In contrast, due to the lack of comparable chronic toxicity data for 6:2 FTSA, two concentrations (50 and 500 µg/L) were chosen to facilitate direct comparison with PFOS and to evaluate any potential dose-dependent effects. Throughout the 30-day experimental period, fish were fed a commercial diet twice daily and housed at 26 ± 1 °C under a 14 h:10 h light-dark cycle.

After treatment, zebrafish were euthanized using MS-222 (200 mg/L, Sigma-Aldrich, St. Louis, MO, USA), followed by immediate dissection on ice to isolate liver tissues. For histological analysis, tissues harvested from three fish per tank were immersion-fixed in 4% paraformaldehyde. Meanwhile, liver samples from two separate sets of three fish per replicate were collected for RNA extraction and MDA measurement, snap-frozen in liquid nitrogen, and stored at −80 °C. The overall study design is illustrated in Figure 1B.

### 2.3. Histological Analysis and MDA Measurement

Histological analysis was performed on paraffin-embedded sections of the fixed liver tissues after hematoxylin and eosin (H&E) staining as previously reported [29]. Hepatic MDA concentration, an indicator of lipid peroxidation, was measured using the thiobarbituric acid (TBA) method according to the kit manufacturer’s guidelines.

### 2.4. Transcriptomic Analysis

To enhance biological replication, liver tissues from three fish within the same tank were pooled to form one composite sample. This sampling strategy yielded three independent biological replicates per treatment group, resulting in a total of 12 samples (CK_1 to CK_3, P50_1 to P50_3, F50_1 to F50_3, and F500_1 to F500_3) for transcriptomic analysis. All subsequent steps, including RNA extraction, library construction and sequencing, were performed by Shanghai Majorbio Bio-pharm Biotechnology Co., Ltd. (Shanghai, China).

All samples exhibited high RNA integrity, with RNA Integrity Numbers (RIN) ≥ 9.0. Sequencing libraries were constructed from 1 μg of total RNA per sample using the Illumina^®^ Stranded mRNA Prep, ligation (San Diego, CA, USA). The main procedures included: mRNA isolation via oligo(dT) beads, RNA fragmentation, double-stranded cDNA synthesis with random primers, adapter ligation, size selection (300–400 bp), and PCR amplification. The resulting libraries were quantified and sequenced on the NovaSeq X Plus platform (PE150) (Illumina, Inc., San Diego, CA, USA).

Raw reads were processed with fastp 0.19.5 to remove adapters and low-quality sequences. The resulting clean reads were aligned to the reference genome (assembly GRCz11, GCA_000002035.4) using HISAT2 2.1.0 with strand-specific settings. Transcript assemblies were then generated for each sample using StringTie 2.1.2 in reference-guided mode.

All subsequent bioinformatic analyses were performed on the Majorbio Cloud Platform. Transcript abundance was quantified using RSEM (in transcripts per million reads, TPM), and differentially expressed genes (DEGs) were identified with DESeq2 1.38.0 (|log_2_(fold change, FC)| ≥ 1, adjusted *p* < 0.05). Functional enrichment analysis for Gene Ontology (GO) terms and Kyoto Encyclopedia of Genes and Genomes (KEGG) pathways was then performed against the whole transcriptome background using Goatools 0.6.5 and SciPy 0.13.3, respectively, with significance set at a Bonferroni-corrected *p* < 0.05. Additionally, KEGG pathway enrichment was assessed via Gene Set Enrichment Analysis (GSEA) following our prior work [30]. A gene set was considered significantly enriched if it met the following criteria: absolute normalized enrichment score (NES) >1 and adjusted *p* < 0.25.

### 2.5. Quantitative Real-Time PCR (qRT-PCR) Validation

To validate the RNA-Seq results, qRT-PCR was performed on a subset of genes using adult female liver tissue (*n* = 3 per group). Total RNA extraction, cDNA synthesis, and qRT-PCR assays were carried out as previously described [29], using *β-actin* as the internal reference gene. All primer sequences are listed in Supplementary Table S1.

### 2.6. Statistics

All data, except for the RNA-seq data, were analyzed by one-way analysis of variance (ANOVA) with Tukey’s post hoc test using GraphPad Prism 8.0.2. Prior to ANOVA, data were confirmed to meet the assumptions of normality by Shapiro–Wilk test and homogeneity of variances by Bartlett’s test. Statistical significance was set at *p* < 0.05.

## 3. Results

### 3.1. PFOS and 6:2 FTSA Induce Histopathological Alterations in Liver

**Figure 2:**
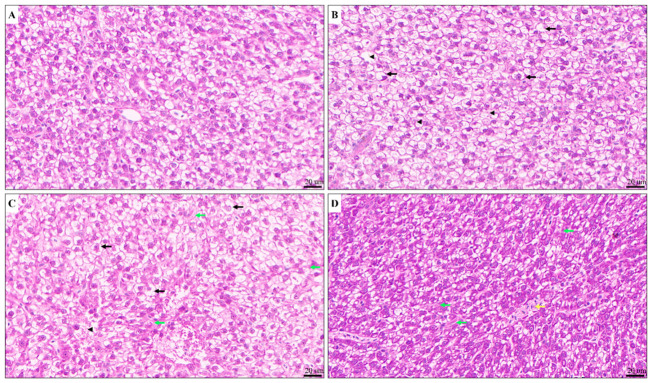
Representative images of hepatic histopathology in zebrafish after exposure to PFOS and 6:2 FTSA. (**A**) Control (CK) liver tissue showing normal liver architecture. (**B**–**D**) Treatment groups display distinct pathological alterations: (**B**) P50 (50 µg/L PFOS), (**C**) F50 (50 µg/L 6:2 FTSA), and (**D**) F500 (500 µg/L 6:2 FTSA). Black arrow: hepatocellular ballooning; black arrowhead: spotty necrosis; green arrow: hepatic fibrosis; yellow arrow: venous congestion. Scale bar: 20 µm.

Histological examination revealed that exposure to PFOS (50 μg/L) or 6:2 FTSA (50 and 500 μg/L) induced histopathological alterations in zebrafish liver (Figure 2). Liver from the control group displayed normal architecture with well-organized hepatocytes. In contrast, exposure to 50 µg/L PFOS or 50 µg/L 6:2 FTSA induced comparable levels of hepatocellular damage, characterized by ballooning degeneration and spotty necrosis. Additionally, livers from the 50 µg/L 6:2 FTSA group exhibited hepatic fibrosis. These fibrotic changes were markedly exacerbated in the 500 µg/L 6:2 FTSA group, which also exhibited venous congestion. These findings indicate a dose-dependent hepatotoxic response to this compound.

### 3.2. Hepatic Transcriptomic Reprogramming Induced by PFOS and 6:2 FTSA

#### 3.2.1. Overview of RNA-Sequencing and Transcriptome Assembly

To delineate the hepatic transcriptomic responses, RNA sequencing was conducted on twelve liver samples, representing four experimental groups in triplicate: solvent control (CK), 50 μg/L PFOS (P50), 50 μg/L 6:2 FTSA (F50), and 500 μg/L 6:2 FTSA (F500). The raw data were deposited in the NCBI database (BioProject ID: PRJNA1455118). A total of 593,460,280 raw reads were obtained. Following quality filtering (Q30 > 95.61%; GC content 45.82–47.29%), about 588,516,460 clean reads were retained. Subsequent ribosomal RNA removal and alignment against the *Danio rerio* reference genome (assembly GRCz11, GCA_000002035.4) yielded high mapping efficiencies, ranging from 88.04% to 90.80% (Table S2). Subsequent genome-guided transcript assembly identified 31,296 genes in total, comprising 25,086 annotated and 6210 novel loci.

#### 3.2.2. Identification of DEGs

**Figure 3:**
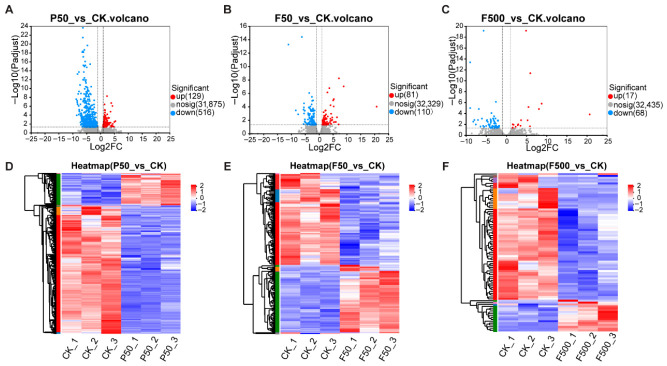
Analysis of differentially expressed genes (DEGs) in adult female zebrafish livers following exposure to PFOS and 6:2 FTSA. (**A**–**C**) Volcano plots showing DEGs for the P50 (**A**), F50 (**B**), and F500 (**C**) groups compared to the control (CK). Red and blue points represent significantly up- and down-regulated genes (|fold change| > 2, adjusted *p* < 0.05), respectively, while grey points indicate non-significant transcripts. (**D**–**F**) Clustered heatmaps displaying the expression patterns of these DEGs across individual samples. Hierarchical clustering was conducted using average linkage and Euclidean distance on row-scaled (Z-score) expression values. The color scale represents expression levels from low (blue) to high (red).

Principal component analysis (PCA) demonstrated clear separation among the treatment groups (Figure S1). Subsequent differential expression analysis under the threshold (|log_2_FC| ≥ 1, adjusted *p* < 0.05) identified 645 DEGs (129 up, 516 down), 191 DEGs (81 up and 110 down), and 85 DEGs (17 up and 68 down) in P50_vs_CK, F50_vs_CK, and F500_vs_CK comparisons, respectively (Figure 3 and Tables S3–S5).

#### 3.2.3. Validation of RNA-Seq Data by qRT-PCR and Insights into Exposure-Specific Responses

**Figure 4:**
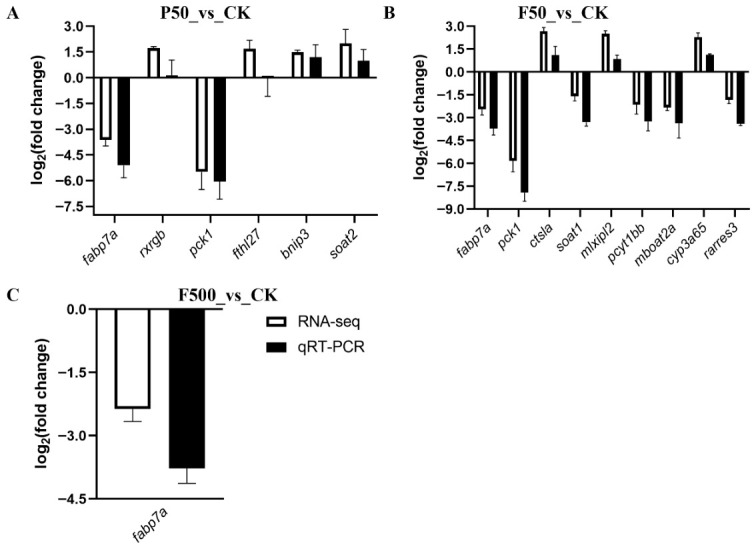
Validation of RNA-seq results by qRT-PCR. Expression changes (log_2_fold change) of selected DEGs are shown as determined by RNA-seq (open bars) and qRT-PCR (filled bars). Data are presented as mean ± SEM (*n* = 3). (**A**) P50_vs_CK comparison. (**B**) F50_vs_CK comparison. (**C**) F500_vs_CK comparison.

The reliability of the RNA-seq data was confirmed by qRT-PCR analysis of selected DEGs (Figure 4). We validated six DEGs (*fabp7a*, *rxrgb*, *pck1*, *fthl27*, *bnip3*, and *soat2*) for the P50_vs_CK comparison, nine DEGs (*fabp7a*, *pck1*, *ctsla*, *soat1*, *mlxipl2*, *pcyt1bb*, *mboat2a*, *cyp3a65*, and *rarres3*) for the F50_vs_CK comparison, and one DEG (*fabp7a*) for the F500_vs_CK comparison. Regression analysis revealed a highly significant positive correlation between the log_2_FC values obtained by the two platforms (R^2^ = 0.9697, *p* < 0.0001), confirming the accuracy of transcriptome sequencing.

Notably, the transcriptional profile induced by the high concentration of 6:2 FTSA (F500) was markedly distinct. Only one gene (*fabp7a*) was both significantly dysregulated and expressed at a robust level (TPM ≥ 10) across all three treatment groups, precluding the construction of a biologically meaningful common validation panel spanning all exposures. This scarcity of shared, high expressed DEGs suggests a fundamental shift in the hepatotoxic mechanism at the high concentration of 6:2 FTSA. Consequently, the focused validation on *fabp7a* for the F500 group reflects this unique transcriptional response rather than a methodological limitation.

#### 3.2.4. Functional Enrichment Analysis of DEGs in GO and KEGG Pathways

**Figure 5:**
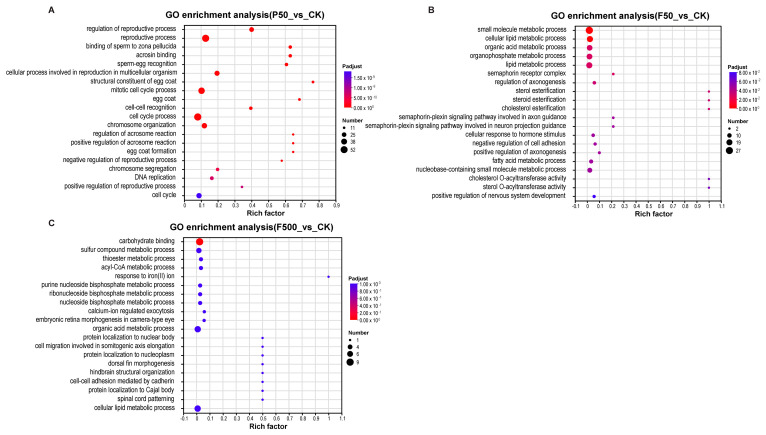
GO enrichment analysis of DEGs. (**A**–**C**) Top 20 enriched functional terms for the comparisons of (**A**) P50_vs_CK, (**B**) F50_vs_CK, and (**C**) F500_vs_CK.

**Figure 6:**
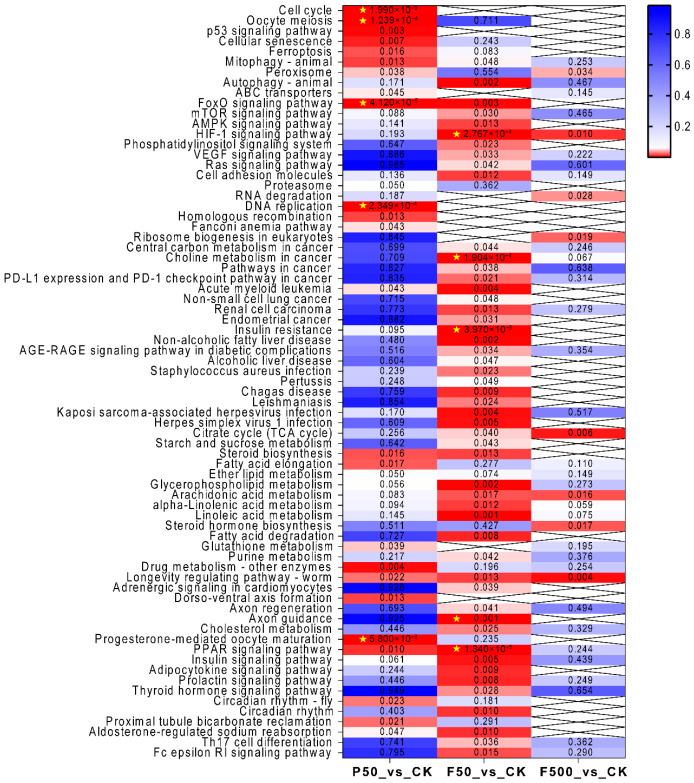
KEGG pathway enrichment analysis of DEGs from the comparisons P50_vs_CK, F50_vs_CK, and F500_vs_CK. The color bar indicates *p* value from low (red) to high (blue). Pathways shown are those that were significantly enriched (*p* < 0.05) in at least one exposure group. A yellow pentagram denotes pathways that remained significant after multiple testing correction (adjusted *p* < 0.05).

To elucidate the biological roles of the identified DEGs, we performed GO and KEGG pathway enrichment analyses (Figure 5 and Figure 6).

In the P50_vs_CK comparison, GO analysis revealed significant enrichment of terms primarily related to reproductive processes and cell cycle regulation. The top enriched GO terms included “regulation of reproductive process”, “binding of sperm to zona pellucida”, “sperm-egg recognition”, “cell cycle process”, “chromosome segregation”, and “DNA replication” (Figure 5A and Table S6). Consistently, KEGG pathway analysis highlighted specific disruptions in “progesterone-mediated oocyte maturation”, “oocyte meiosis”, and the “FoxO signaling pathway”. Additional enrichment was observed in pathways governing genotoxic stress and DNA damage response, such as the “cell cycle”, “DNA replication”, “p53 signaling pathway” and “homologous recombination”, alongside metabolic pathways like “PPAR signaling pathway” and “steroid biosynthesis” (Figure 6 and Table S9).

For the F50_vs_CK comparison, GO results demonstrated a strong emphasis on metabolic processes, particularly lipid and sterol homeostasis—evidenced by terms such as “cellular lipid metabolic process”, “fatty acid metabolic process”, “sterol esterification”, “steroid esterification”, and “cholesterol esterification” (Figure 5B and Table S7). This metabolic shift was further supported by KEGG analysis, where the most significantly enriched pathways included “PPAR signaling pathway”, “insulin resistance”, and “non-alcoholic fatty liver disease”. Other notable pathways, such as “linoleic acid metabolism”, “glycerophospholipid metabolism”, and nutrient-sensing cascades (e.g., “AMPK signaling pathway” and “mTOR signaling pathway”), reinforced the pervasive reprogramming of hepatic metabolism (Figure 6 and Table S10).

In the F500_vs_CK comparison, GO analysis identified only one significantly enriched term, “carbohydrate binding”, following multiple-testing correction (adjusted *p* < 0.05) (Figure 5C and Table S8). Correspondingly, KEGG analysis indicated limited pathway perturbations, with the most noticeable—though not statistically significant after adjustment—changes occurring in core energy metabolism pathways such as the “citrate cycle (TCA cycle)” and several lipid metabolic processes (Figure 6 and Table S11).

#### 3.2.5. Enriched KEGG Pathways Using GSEA

**Figure 7:**
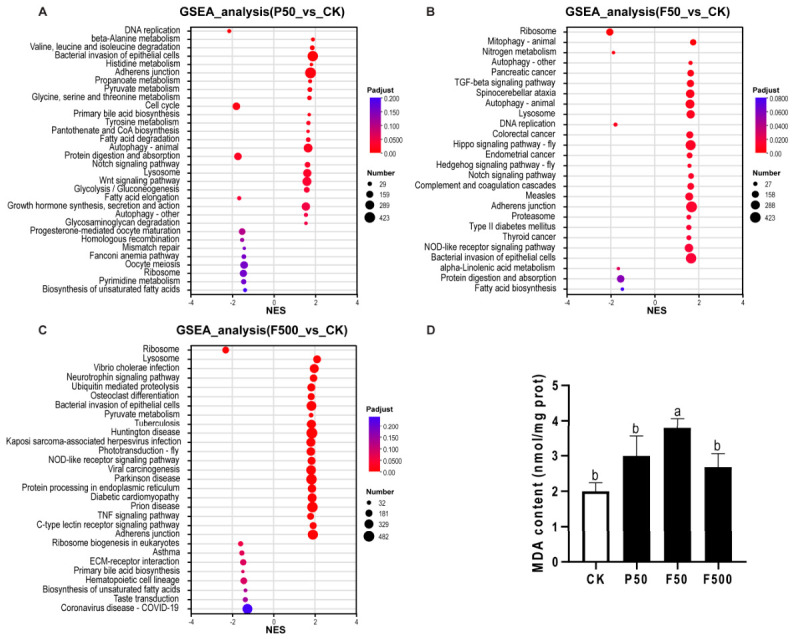
GSEA of enriched KEGG pathways (**A**–**C**) and assessment of hepatic lipid peroxidation (**D**). (**A**–**C**) Heatmap showing KEGG pathways significantly enriched (|NES| > 1 and adjusted *p* < 0.25). Color indicates the adjusted *p*-value from low (red) to high (blue). Abbreviations: NES, normalized enrichment score; Number, core gene number; Padjust, adjusted *p*-value. (**D**) Hepatic MDA levels in zebrafish after exposure to PFOS or 6:2 FTSA. Data shown are mean ± SEM (*n* = 3). Bars marked with different letters are significantly different (*p* < 0.05).

To further elucidate the biological mechanisms and complement conventional DEG analysis, KEGG pathways were analyzed using GSEA across the three comparative groups (Figure 7A–C and Table S12). In the PFOS-exposed group (P50), cellular proliferation-related pathways (e.g., “DNA replication”, “cell cycle”, “homologous recombination”) were significantly downregulated, while pathways involved in metabolic reprogramming (e.g., “beta-Alanine metabolism”, “pyruvate metabolism”, “glycolysis/gluconeogenesis”) and cell stress (“autophagy—animal”, “lysosome”) were significantly upregulated. Following low-dose 6:2 FTSA exposure (F50), pathways such as “Ribosome” and “DNA replication” were significantly downregulated, whereas pathways related to cellular stress (e.g., “mitophagy—animal”, “lysosome”) and inflammation/immunity (e.g., “complement and coagulation cascades”, “NOD-like receptor signaling pathway”) were significantly upregulated. In the high-dose 6:2 FTSA group (F500), “ribosome” was significantly downregulated, while pathways including “lysosome”, “protein processing in endoplasmic reticulum”, and “ubiquitin mediated proteolysis” were significantly upregulated.

### 3.3. Effects of PFOS and 6:2 FTSA on MDA Levels in Liver

Hepatic MDA levels were significantly elevated (*p* < 0.05) in zebrafish exposed to 50 µg/L 6:2 FTSA compared to the control. In contrast, no significant changes were observed in the groups treated with 50 µg/L PFOS or 500 µg/L 6:2 FTSA (Figure 7D).

## 4. Discussion

Globally, long-chain PFASs, notably PFOS, face stringent regulatory controls owing to their well-documented persistence, bioaccumulative potential, and toxicity. Consequently, PFOS is being progressively phased out from most commercial applications, driving the widespread adoption of short-chain alternatives such as 6:2 FTSA. This alternative is now frequently detected in various environmental matrices, including aquatic systems. With expanding industrial applications and inherent environmental persistence, both the detection frequency and environmental concentrations of 6:2 FTSA are expected to rise persistently, marking it as a contaminant of emerging global concern. Given the structural similarity between PFOS and 6:2 FTSA, we posited that the latter would also induce hepatotoxicity in zebrafish. However, systematic investigations examining the chronic hepatic effects of 6:2 FTSA, particularly in direct comparison with PFOS, remain notably lacking. To address this knowledge gap, we conducted a 30-day chronic exposure experiment using adult female zebrafish to control for sexual dimorphism and to define the female-specific hepatic response. Experimental groups were exposed to 50 μg/L PFOS, 50 μg/L 6:2 FTSA, and 500 μg/L 6:2 FTSA. Our integrated findings demonstrate that 6:2 FTSA is not a safe alternative to PFOS but exhibits a dose-dependent and mechanistically shifting hepatotoxic profile, with actions that diverge from its long-chain counterpart.

### 4.1. Effects of PFOS and 6:2 FTSA on Liver Histopathology

Fish histopathology constitutes an essential methodology in aquatic toxicological assessment. PFOS exposure is a well-established cause of hepatic injury in zebrafish [24,25,28,31,32], with some studies noting greater severity in males [25,28,32]. Nevertheless, in our 30-day exposure study using adult female zebrafish, exposure to 50 μg/L 6:2 FTSA induced histopathological alterations—including ballooning degeneration and spotty necrosis—that were comparable to those caused by PFOS at the same concentration, with the additional feature of hepatic fibrosis. Although no prior studies document 6:2 FTSA-induced hepatotoxicity in zebrafish, chronic exposure in adult male mice has been shown to promote increased liver weight, accompanied by inflammatory infiltration and necrotic alterations [26], thereby identifying the liver as a vulnerable target organ. These morphological observations align with the established hepatotoxic profile of PFOS in zebrafish [25,32]. Moreover, the hepatotoxic effects demonstrated a clear dose–response relationship, as 500 μg/L 6:2 FTSA provoked substantially aggravated liver injury, marked by intensive eosinophilic staining, severe disruption of hepatic lobular architecture, and extensive parenchymal necrosis. Collectively, these findings indicate that 6:2 FTSA imposes considerable health risks on aquatic organisms. Consequently, the presumption of 6:2 FTSA as a safe alternative to PFOS warrants thorough toxicological scrutiny.

### 4.2. Effects of PFOS and 6:2 FTSA on the Number of Hepatic DEGs

In this study, hepatic transcriptomic profiling was employed to delineate the molecular response patterns in zebrafish following exposure to PFOS and its short-chain alternative, 6:2 FTSA. Comparative analysis identified 645, 191, and 85 DEGs for the P50_vs_CK, F50_vs_CK, and F500_vs_CK comparisons, respectively. These quantitative distinctions clearly demonstrate that under identical exposure conditions (50 μg/L, 30 days), PFOS elicits a significantly broader disruption of hepatic gene expression than 6:2 FTSA. This finding aligns with reports in other organisms, such as earthworms (*Eisenia fetida*), where 6:2 FTSA exhibited lower toxicity than PFOS without provoking substantial transcriptome-wide alterations [33]. The substantial difference in DEG numbers suggests fundamental differences in their molecular mechanisms of action, a notion supported by toxicokinetic studies in female mice showing higher hepatic accumulation of PFOS compared to 6:2 FTSA [34].

More strikingly, the high-concentration F500 group displayed a substantially lower DEG count (*n* = 85) compared to the F50 group (*n* = 191), despite exhibiting the most severe histopathological injury (Section 3.1). To rule out technical artifacts, we confirmed that RNA integrity (RIN ≥ 9.0) and yield were high and comparable across all groups, indicating that the transcriptomic data robustly reflect the in vivo biological state. We interpret this pattern not as an attenuated biological response, but as a reflection of a fundamental, dose-dependent mechanistic shift: from metabolic perturbation (F50) to overwhelming cellular stress and injury (F500).

### 4.3. P50_vs_CK Transcriptomics Reveals Profound Disruption of Cell Proliferation and Reproduction

In our comparative analysis, exposure to 50 μg/L PFOS (P50 group) induced the most pronounced transcriptomic alterations. These changes were dominated by the widespread suppression of cell cycle and DNA replication processes, as clearly demonstrated by GSEA. This finding is consistent with earlier in vitro studies in zebrafish liver cell lines (ZFL), where PFOS exposure led to cell cycle arrest and altered expression of associated genes [35]. Such disruption of core proliferative mechanisms suggests a genotoxic potential of PFOS, possibly via activation of damage-response pathways such as p53 [36], which could ultimately impair hepatic regenerative and repair capacity.

Moreover, GO and KEGG enrichment analyses strongly indicated reproductive toxicity. Enrichment of pathways including “progesterone-mediated oocyte maturation”, “oocyte meiosis”, and several oocyte membrane formation-related terms suggested that PFOS severely disrupts hepatic functions linked to female reproduction. As the primary site for vitellogenin (Vtg) synthesis, a protein essential for oocyte development [37], the liver appears to be a key target. These results align with previous reports identifying PFOS as an endocrine disruptor that impairs reproductive health [38]. The disturbed reproduction-related pathways observed in female fish liver further support the proposed mechanism by which PFOS interferes with the liver–gonad axis to compromise reproduction, consistent with related findings in male zebrafish [27].

Notably, while our transcriptomic analysis did identify subtle perturbations in lipid metabolism pathways in PFOS-exposed fish (e.g., a nominal enrichment of the PPAR signaling pathway in KEGG analysis, *p* < 0.05, and a significant enrichment of the “Biosynthesis of unsaturated fatty acids” pathway in GSEA), the scale and signature of this disruption were markedly different from the robust, PPAR-centric metabolic reprogramming induced by 50 μg/L 6:2 FTSA (Section 4.4). The relatively weak lipid metabolism signature for PFOS, compared to the strong PPAR-driven response for 6:2 FTSA, is likely attributable to key experimental conditions. First, the use of adult female zebrafish is a critical factor. Proteomic studies have established that zebrafish liver exhibits fundamental sex-specific differences in the abundance of proteins involved in lipid metabolism [39]. Second, the 30-day exposure at 50 µg/L may represent a condition where the primary and immediate toxic insult is direct cytotoxicity (cell cycle arrest), potentially preceding or overshadowing a full-scale adaptive metabolic response. Therefore, while both compounds can affect lipid-related pathways, direct mechanistic comparisons should be nuanced. Our data thus delineate a clear mechanistic distinction: PPAR-mediated metabolic disruption constitutes the central and defining mode of action for 6:2 FTSA hepatotoxicity, whereas PFOS hepatotoxicity under these experimental conditions is primarily characterized by a cytotoxic (cell cycle arrest) and reproductive toxic response, with lipid metabolism perturbation representing a secondary component. Additionally, significant enrichment of the “FoxO signaling pathway” in KEGG analysis, along with upregulation of “autophagy” and “lysosome” pathways detected by GSEA, indicates that cells activate stress defense and metabolic reprogramming in response to PFOS-induced toxicity.

### 4.4. F50_vs_CK Transcriptomics Reveals PPAR-Mediated Metabolic Disruption by Low-Dose 6:2 FTSA

In contrast, exposure to 50 μg/L 6:2 FTSA (F50 group) caused comparatively milder transcriptomic alterations via a distinct mechanism, primarily involving PPAR pathway-mediated metabolic disruption. GO analysis revealed significant enrichment of DEGs in processes such as “cellular lipid metabolic process”, “fatty acid metabolic process”, and “steroid esterification”. This pattern indicates a specific disruption of hepatic lipid and sterol homeostasis, with potential downstream effects on steroid hormone signaling. KEGG analysis further corroborated this, showing marked enrichment of the “PPAR signaling pathway”, a key regulator of fatty acid oxidation and lipid transport [40]. This suggests that 6:2 FTSA likely acts as a PPAR ligand, driving extensive lipid metabolic reprogramming, consistent with reports on other PFAS alternatives such as F-53B [41]. The “insulin resistance” pathway enrichment also implies disrupted glucose metabolism, potentially elevating metabolic disorder risks.

GSEA indicated upregulation of “mitophagy–animal” and “lysosome” pathways, suggesting mitochondrial damage and activation of quality control mechanisms. Additionally, enhanced activity in pathways such as “complement and coagulation cascades” and “NOD-like receptor signaling pathway” aligns with reported immunotoxicity in zebrafish embryos [18]. Notably, the F50 group also showed downregulation of “ribosome” and “DNA replication” pathways, indicating a certain degree of suppression in protein synthesis and cell proliferation. However, this suppression was far less pronounced than that observed in the P50 group, suggesting that the genotoxic potential of 6:2 FTSA is relatively weak.

### 4.5. F500_vs_CK Transcriptomics Reveals a Dose-Dependent Mechanistic Shift Toward Acute Cellular Stress and Injury

The transcriptomic profile of the high-dose 6:2 FTSA group (F500) revealed a pivotal shift in the mechanism of toxicity. Despite the most severe histopathology, this group showed fewer DEGs. This apparent discrepancy is reconciled by GSEA, which revealed a focused but intense upregulation of pathways related to endoplasmic reticulum (ER) stress (“protein processing in endoplasmic reticulum”), proteolysis (“ubiquitin mediated proteolysis”), and lysosomal function. Concurrently, there was a pronounced global downregulation of biosynthetic and metabolic pathways, most notably “ribosome”. This pattern indicates a fundamental mechanistic shift at high dose: from the metabolic dysregulation seen at F50 to a state of acute proteotoxic crisis and cellular injury at F500. Under such severe stress, hepatocytes likely prioritize emergency response pathways while broadly suppressing other transcriptional programs, a phenomenon potentially compounded by a loss of transcriptional activity in necrotic cells. This explains the reduced DEG count not as reduced toxicity, but as a reflection of a transcriptomic landscape dominated by a focused stress response and global functional repression. This interpretation aligns with the concept of a dose-dependent mechanistic shift, where the primary toxic insult changes with increasing concentration.

### 4.6. The Distinct Pattern of MDA Response Supports Differential Toxicity Mechanisms of PFOS and 6:2 FTSA

This study elucidated distinct toxic mechanisms for PFOS and 6:2 FTSA through transcriptomic analysis, with critical biochemical context provided by assessing MDA, a key marker of lipid peroxidation [42]. A notable finding was the pattern of hepatic MDA levels: a significant elevation was observed only in zebrafish exposed to 50 μg/L 6:2 FTSA (F50 group). In contrast, neither the PFOS-exposed group (P50) at an equivalent concentration nor the high-dose 6:2 FTSA group (F500) showed a significant change.

This biochemical pattern provides supportive evidence for the transcriptomic findings. The marked rise in MDA within the F50 group is consistent with its “metabolic toxicity” phenotype, characterized by PPAR pathway activation and disrupted lipid metabolism. Conversely, the primary toxic action of PFOS (P50) likely involves direct inhibition of the cell cycle and DNA repair pathways. Its effects likely manifest earlier as proliferative arrest rather than through widespread oxidative stress, potentially explaining the absence of a significant MDA increase.

The lack of elevated MDA in the F500 group is unexpected. One plausible explanation, consistent with the transcriptomic data, is a shift towards injury pathways less reliant on lipid peroxidation, such as direct proteotoxicity and ER stress, at the higher concentration. This interpretation is offered cautiously, and the precise mechanism warrants further investigation.

### 4.7. Study Implications and Limitations

This study provides integrated evidence that 6:2 FTSA induces potent hepatotoxicity via mechanisms distinct from PFOS, challenging its status as a safe alternative. Several considerations should be noted when interpreting these findings. First, the exclusive use of female zebrafish, while controlling for sex-based variance and providing crucial baseline data for an understudied group, limits insight into potential sex-dependent effects and may include variability from hormonal cycles. Second, the selected exposure concentrations, while critical for revealing the dose-dependent mechanistic shift and distinct mechanisms of 6:2 FTSA, are higher than typical environmental levels; future studies at environmentally relevant doses are essential for refined risk assessment. Third, the transcriptomic predictions, particularly regarding PPAR-mediated disruption by 6:2 FTSA and the severe stress response at high dose, are primarily hypothesis-generating. Claims about lipid metabolic dysfunction and cellular injury would be strengthened by complementary functional endpoints such as hepatic triglyceride/cholesterol quantification, histopathological lipid staining (e.g., Oil Red O), or serum transaminase (ALT/AST) levels. The absence of such biochemical validation is acknowledged as a study limitation. Future work incorporating these metrics is essential to functionally confirm the mechanistic pathways proposed here. Notwithstanding these limitations, the distinct and dose-complex hepatotoxicity profile of 6:2 FTSA uncovered here necessitates a reevaluation of its environmental safety.

## 5. Conclusions

In summary, this study systematically delineates the differential hepatotoxicity mechanisms of legacy PFOS and its emerging alternative, 6:2 FTSA, following chronic exposure in adult female zebrafish. We demonstrate that these compounds pose distinct hazards rather than representing a straightforward substitution. PFOS exposure (50 μg/L) acted as a potent disruptor of core biological processes, primarily impairing cell proliferation and reproduction. In contrast, 6:2 FTSA exhibited a more complex, dose-dependent toxicity. At a lower concentration (50 μg/L), it primarily dysregulated metabolic homeostasis via PPAR-mediated pathways. At a higher concentration (500 μg/L), it triggered a fundamental shift towards acute proteotoxic and ER stress, leading to severe cellular injury and a focused, repressive transcriptomic response. Notably, the distinct pattern of lipid peroxidation (MDA) further confirms the differing mechanistic underpinnings.

Our findings challenge the presumption of 6:2 FTSA as a safe alternative and reveal the limitation of substitution strategies based merely on structural analogy. Evaluating such emerging pollutants requires testing across a broad dose range and monitoring for compound-specific toxicity endpoints. Future research should prioritize: (i) direct functional validation (e.g., via Oil Red O staining or triglyceride quantification) of the predicted lipid metabolism disorders; (ii) experiments in male zebrafish and at environmentally relevant concentrations to assess sex-specific and real-world risks; and (iii) proteomic validation of the proposed pathways and investigation into long-term and transgenerational effects. These steps are essential to fully assess the ecological risks of PFAS alternatives.

## Data Availability

Data will be made available on reasonable request.

## Supplementary Materials

The following supporting information can be downloaded at: https://www.mdpi.com/article/10.3390/ani16091368/s1, Figure S1: Principal component analysis (PCA) of the hepatic transcriptomic profile; Table S1: Primer sequences for amplification of target genes in this study; Table S2: Summary of transcriptome sequencing; Table S3: The DEGs in P50_vs_CK (fold change > 2 and adjusted *p* < 0.05); Table S4: The DEGs in F50_vs_CK (fold change > 2 and adjusted *p* < 0.05); Table S5: The DEGs in F500_vs_CK (fold change > 2 and adjusted *p* < 0.05); Table S6: GO terms enrichment in P50_vs_CK (*p* < 0.05); Table S7: GO terms enrichment in F50_vs_CK (*p* < 0.05); Table S8: GO terms enrichment in F500_vs_CK (*p* < 0.05); Table S9: KEGG pathways enrichment in P50_vs_CK; Table S10: KEGG pathways enrichment in F50_vs_CK; Table S11: KEGG pathways enrichment in F500_vs_CK; Table S12: The up- and down-regulated pathways by GSEA-KEGG pathways analysis (|NES| > 1, adjusted *p* < 0.25).
