# Supplementary material for: Comparative Hepatotoxicity Assessment of PFOS and Its Alternative 6:2 FTSA in Adult Female Zebrafish
Source: Animals (Basel). 2026 Apr 29;16(9):1368. doi: 10.3390/ani16091368 (PMC13162690; doi:10.3390/ani16091368)
Supplement: Supplementary file 1 [file animals-16-01368-s001.zip › Figure S1.pdf]

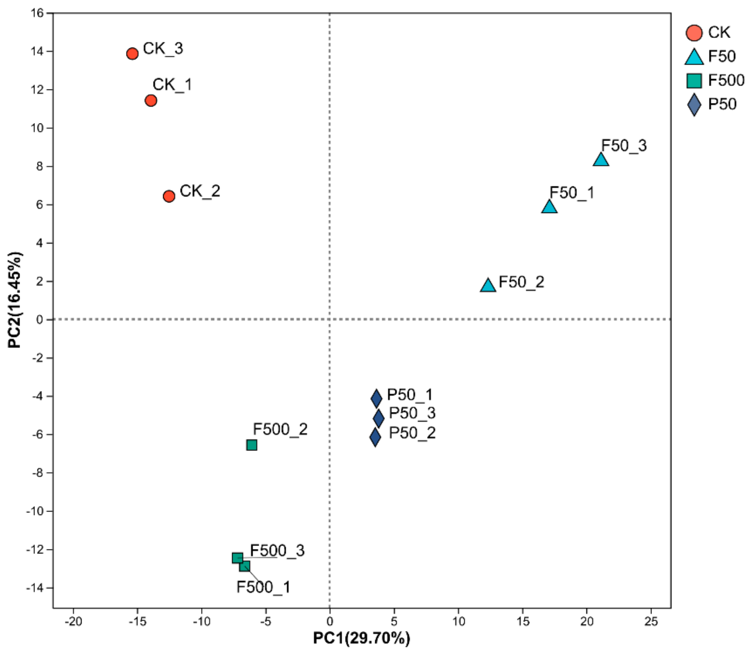

Figure S1. Principal component analysis (PCA) of the hepatic transcriptomic profile. The plot depicts global gene expression patterns in liver samples from adult female zebrafish across different treatment groups: control (CK), 50 µg/L PFOS (P50), 50 µg/L 6:2 FTSA (F50), and 500 µg/L 6:2 FTSA (F500). Each point corresponds to an individual biological replicate.
